# Shikonin-Loaded Nanoparticles Ameliorate DEHP-Exacerbated Psoriasis Skin Injury via Targeted Inhibition of the p38 MAPK Signaling Pathway

**DOI:** 10.3390/ijms27167109

**Published:** 2026-08-08

**Authors:** Qinghua Tang, Yixiong Li, Yan Li, Xinyuan Wang, Yanan Bie, Xuesong Yu, Lin Zhou, Ming Li

**Affiliations:** 1 School of Basic Medical Sciences, Guangdong Pharmaceutical University, Guangzhou Higher Education Mega Centre, Guangzhou 510006, China; 2112440144@stu.gdpu.edu.cn (Q.T.); 2112440139@stu.gdpu.edu.cn (Y.L.); bieyanan@gdpu.edu.cn (Y.B.); 2 School of Basic Medical Sciences, Guangzhou University of Chinese Medicine, Guangzhou Higher Education Mega Centre, Guangzhou 510006, China; liy506@gzucm.edu.cn (Y.L.); xinyuan.wang@gzucm.edu.cn (X.W.); 3 School of Life Science and Biopharmaceutics, Guangdong Pharmaceutical University, Guangzhou Higher Education Mega Centre, Guangzhou 510006, China; yuxuesong@gdpu.edu.cn

**Keywords:** di-(2-ethylhexyl) phthalate (DEHP), psoriasis, shikonin nanoparticles, p38 MAPK pathway, inflammatory microenvironment

## Abstract

Di-(2-ethylhexyl) phthalate (DEHP), a ubiquitous environmental plasticizer, has been increasingly linked to the exacerbation of inflammatory skin conditions, especially psoriasis. However, the mechanisms and effective therapeutic strategies targeting DEHP-aggravated psoriasis remain elusive. We integrated network toxicology, network pharmacology, and molecular docking to explore the targets of DEHP-exacerbated psoriasis and the protective effects of shikonin (SH). These findings were validated in a DEHP/IMQ-induced mouse model using a novel shikonin nanoparticle (SH-NP) that overcomes SH’s inherent hydrophobicity. Computational analyses revealed that SH counteracts DEHP skin toxicity through a multi-target network, identifying the p38 mitogen-activated protein kinase (p38 MAPK), TNF, IL-1β, proliferating cell nuclear antigen (PCNA), and matrix metalloproteinases 2 and 9 (MMP2/9) as core therapeutic nodes. Molecular docking verified this network, revealing robust binding between SH and key targets with binding energies ranging from −6.0 to −7.8 kcal/mol, consistently outperforming DEHP. In vivo experiments demonstrated that topical application of SH-NP significantly ameliorated macroscopic skin injury and reduced PASI scores. Mechanistically, SH-NP effectively reversed the DEHP-exacerbated inflammatory microenvironment by decreasing macrophage and mast cell infiltration in the dermis, reducing pro-inflammatory cytokine levels, and inhibiting the phosphorylation of p38 MAPK. Furthermore, SH-NP treatment successfully suppressed keratinocyte hyperproliferation, as evidenced by downregulated PCNA expression and reduced epidermal thickness. SH-NPs also markedly downregulated the elevated levels of MMP2/9 in mice, thereby mitigating collagen degradation and maintaining dermal matrix integrity. This study shows that SH-NPs alleviate DEHP-aggravated psoriasis by suppressing local inflammation, keratinocyte hyperproliferation, and collagen degradation, offering a targeted nanostrategy for environmentally exacerbated skin diseases.

## 1. Introduction

Psoriasis is a common chronic, immune-mediated inflammatory dermatosis, affecting approximately 2–3% of the global population [1]. It is characterized by scaly, erythematous plaques or patches that impair skin esthetics and affect the patient’s daily life, work, mental health, social interactions, and major life decisions [2]. Pathologically, it is characterized by the hyperproliferation of keratinocytes, infiltration of immune cells and production of inflammatory factors [3]. Currently, the precise etiology of psoriasis has not been fully elucidated, and no curative treatment exists.

Accumulating evidence suggests that the toxic effects of plasticizers, as environmental pollutants, on the skin and immune system may trigger or exacerbate psoriasis [4]. Recently, attention has focused on di-(2-ethylhexyl) phthalate (DEHP), one of the most prevalent phthalate plasticizers widely utilized in plastic products, medical devices, and personal care items [5]. Since DEHP is found in so many everyday products, human exposure is almost unavoidable, which is why its metabolic byproducts are commonly detected in urine, blood, and breast milk [6]. An epidemiological study based on NHANES data provided the first population-level evidence showing that psoriasis patients have significantly higher levels of DEHP byproducts in their urine compared to healthy individuals [7]. Supporting this clinical relevance, comprehensive meta-analyses have further demonstrated that cumulative DEHP exposure significantly elevates the risk of related chronic inflammatory skin diseases, while concurrently triggering systemic immune dysregulation [8]. Importantly, while baseline DEHP exposure alone has been shown to induce epidermal barrier disruption and mild cutaneous toxicity [9], subjects with pre-existing psoriasis exhibit a significantly heightened sensitivity to DEHP-exacerbated inflammation and lesion exacerbation [10]. This profound differential susceptibility underscores the clinical rationale for specifically investigating the combined, synergistic effects of DEHP exposure within the context of psoriasis. However, despite this growing evidence, the precise molecular mechanisms by which DEHP hijacks these signaling axes to worsen psoriasis remain incompletely understood. Furthermore, there is currently no specific treatment strategy for psoriasis exacerbated by DEHP exposure, highlighting an urgent need for further mechanistic and therapeutic exploration.

The pathogenesis of psoriasis involves a complex interaction between hyperproliferative keratinocytes and dysfunctional immune cells [11]. Specifically, the profound infiltration of inflammatory cells, including macrophages and mast cells, into the dermis and epidermis constitutes a critical pathological hallmark [12,13]. These infiltrated immune cells continuously secrete large amounts of pro-inflammatory cytokines, notably tumor necrosis factor-α (TNF-α) and interleukin-1β (IL-1β), which maintain and amplify the local inflammatory microenvironment. TNF-α not only directly promotes the abnormal proliferation of keratinocytes but also activates key downstream inflammation signaling cascades. Among them, the p38 mitogen-activated protein kinase (p38 MAPK) pathway, acting as a vital hub connecting extracellular inflammatory signals to intracellular responses, is essential in the occurrence and development of psoriasis [14]. The activation of p38 MAPK further drives the production of inflammatory mediators [15,16]. Consistent with this, studies have demonstrated that TNF-α levels are significantly elevated in the serum and skin injuries of psoriasis patients, inducing a marked upregulation of p38 MAPK phosphorylation in the affected tissues [17]. Targeted inhibition of the p38 MAPK pathway has been proven to effectively break this vicious inflammatory cycle and attenuate psoriasis [18].

In addition to inducing inflammatory factor expression, activation of p38 MAPK can exacerbate epidermal hyperplasia by driving abnormal expression of proliferating cell nuclear antigen (PCNA). As a key marker of keratinocyte hyperproliferation, PCNA is highly expressed in psoriatic injury, and its effective inhibition is directly associated with the alleviation of psoriasis skin injury [19,20]. Furthermore, elevated local expression of MMPs in psoriasis is also tightly associated with the activation of the p38 MAPK pathway. The MMP family is a class of enzymes associated with tissue remodeling and inflammatory infiltration. Research indicates that in the IMQ-induced psoriasis mouse model, levels of MMP-2 and MMP-9 are significantly increased in inflamed skin [21]. In psoriasis patients, serum MMP-9 levels are significantly higher than in healthy controls, and TNF-α can stimulate monocytes to upregulate the expression of various MMPs [22].

Shikonin (SH) is a natural compound extracted from the roots of the *Lithospermum erythrorhizon* plant. It offers several biological benefits, which include fighting inflammation, balancing the immune system, and protecting cells from oxidative stress [23]. Previous research has demonstrated that SH can alleviate tissue damage in various inflammatory diseases by inhibiting the activation of the MAPK signaling pathway [24]. Chi et al. also reported that SH treatments could ameliorate psoriasis skin injury in IMQ-induced mouse models [25]. Although recent bioinformatic analyses predict that SH directly targets essential molecules like MAPK to halt disease progression [26], these computational findings still lack comprehensive in vivo experimental validation. Furthermore, the severe hydrophobicity and poor aqueous solubility of SH significantly limit its bioavailability, thereby hindering its widespread research and clinical application. To overcome this critical bottleneck, nano-formulations offer a highly effective strategy to enhance drug dissolution and cellular uptake. With the advancement of nanotechnology, an increasingly diverse array of nanomedicines has been widely applied, particularly in disease treatment [27,28,29]. Therefore, an SH-NP formulation was utilized in the present study for the subsequent in vivo validation of the predicted pharmacological mechanisms.

Network toxicology, as a holistic analytical approach, aims to decipher complex “pollutant–target–disease” interactomes, enabling the systematic identification of key signaling hubs and biological pathways involved in chemically induced toxicity [30]. Network pharmacology is a method based on systems biology that can systematically predict the active ingredients, targets, and signaling pathways of natural products [31]. Network pharmacology effectively explains how traditional Chinese medicines work through a “multi-component, multi-target” mechanism. By combining network toxicology with network pharmacology, it is hypothesized to identify the pathogenic targets of toxins and the potential therapeutic effects of corresponding drugs [32].

Therefore, in the present study, a comprehensive approach integrating network toxicology, network pharmacology, and molecular docking was employed to explore the pathological mechanisms of DEHP-exacerbated psoriasis and the potential protective action of SH. Subsequently, a psoriasis-like mouse model was established via a combination of DEHP and IMQ, and the mice were further treated with SH-NP to validate the bioinformatic predictions. This research provides new experimental evidence for psoriasis aggravated by environmental pollutants and its treatment with natural active compounds, while also laying the groundwork for the clinical use of SH-NP as a potential therapeutic agent to combat psoriasis.

## 2. Results

### 2.1. Identification of Core Targets and Functional Enrichment Analysis of Shikonin Intervention in DEHP-Exacerbated Psoriasis

**Figure 1:**
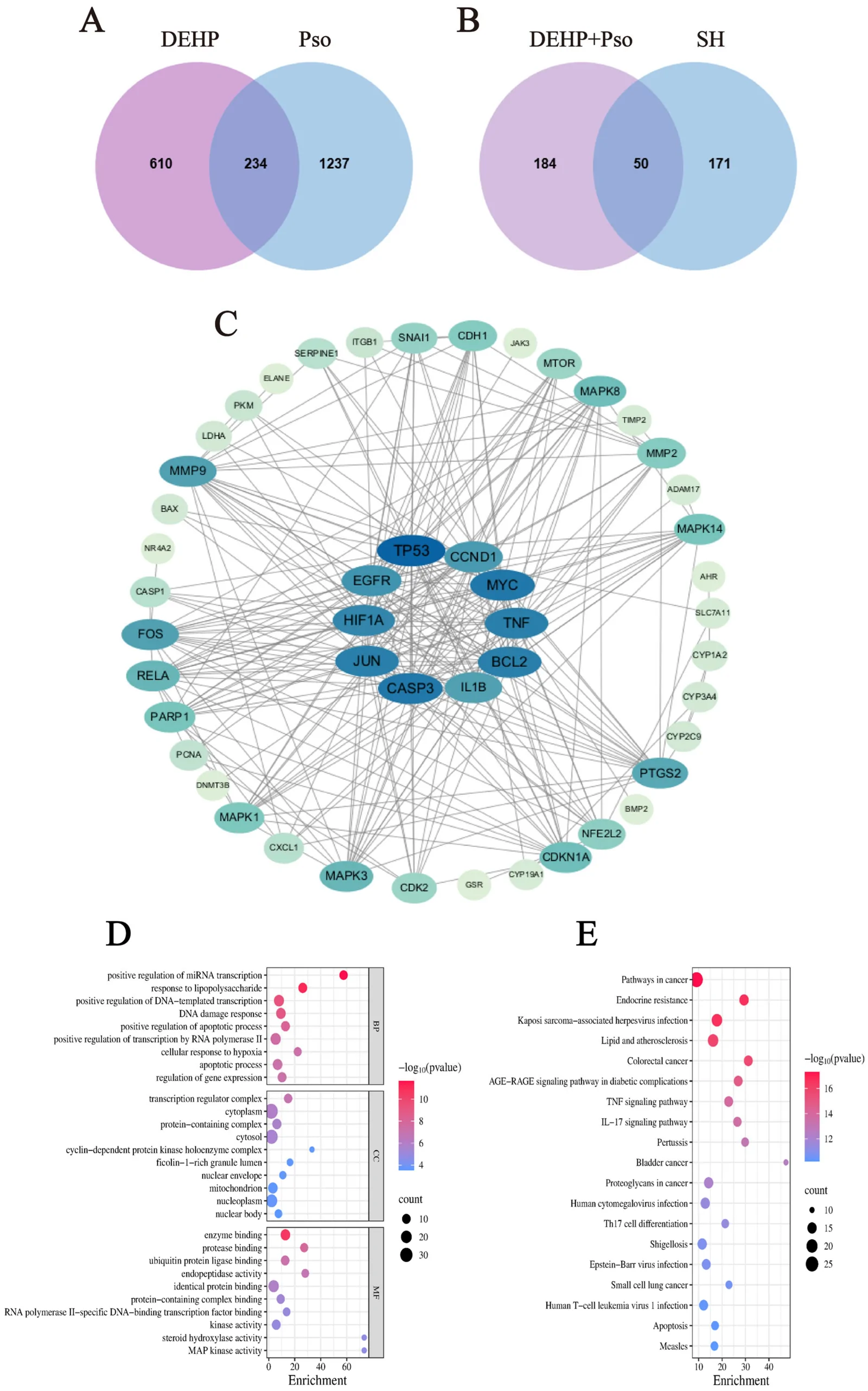
Target identification, protein–protein interaction (PPI) network construction, and functional enrichment analysis. (**A**) Venn diagram illustrating the intersecting targets between DEHP and psoriasis. (**B**) Venn diagram showing the overlapping targets between the DEHP–psoriasis intersection and SH. (**C**) PPI network of the core intersecting targets. Node size and color intensity are proportional to their topological degree in the network, with central dark blue nodes representing hub genes. (**D**) Gene Ontology (GO) enrichment analysis of the core targets, categorized into Biological Process (BP), Cellular Component (CC), and Molecular Function (MF). (**E**) Kyoto Encyclopedia of Genes and Genomes (KEGG) pathway enrichment analysis of the core targets. In panels (**D**,**E**), the color gradient indicates the statistical significance (−log10 *p*-value), and the bubble size represents the number of enriched genes (count).

Network toxicology was used to identify the potential toxic targets of DEHP-exacerbated psoriasis. A total of 844 DEHP-related targets were obtained from SwissTargetPrediction, ChEMBL, and CTD, alongside 1471 psoriasis-related targets retrieved from GeneCards, OMIM, and DrugBank. The intersection of these two sets yielded 234 common targets (Figure 1A), suggesting that DEHP primarily exerts its toxicological and disease-exacerbating effects on psoriasis through these shared biological targets.

Additionally, 221 unique SH-related targets were retrieved using PharmMapper, SwissTargetPrediction, and the CTD database. To identify the specific mechanisms by which SH intervenes in DEHP’s skin toxicity, we mapped these drug targets onto the previously identified 234 common targets of DEHP and psoriasis. This intersection revealed 50 overlapping genes shared among the toxicant, the disease, and the therapeutic drug (Figure 1B), which were designated as the core candidate targets for SH intervention in DEHP-exacerbated psoriasis.

PPI network analysis was conducted by importing the 50 core targets into the STRING database, with subsequent visualization and analysis using Cytoscape (Version 3.10.3). The resulting network exhibited a highly interconnected architecture, suggesting a synergistic regulatory role among these proteins (Figure 1C). Based on topological parameters, the top-ranked hub genes identified included TP53, CCND1, MYC, EGFR, HIF1A, TNF, JUN, BCL2, CASP3, and IL-1β. In addition to these primary hubs, multiple members of the mitogen-activated protein kinase family such as MAPK1, MAPK3, MAPK8, and MAPK14, alongside matrix metalloproteinases including MMP2 and MMP9, emerged as central functional nodes within the network. The prominent positioning and high connectivity of these MAPK and MMP family proteins strongly underscore their essential roles in the molecular mechanisms targeted by SH-NPs in DEHP-related psoriasis.

To further elucidate the biological significance and underlying regulatory mechanisms of these core targets, systematic GO functional annotation and KEGG pathway enrichment analyses were performed via DAVID. In terms of the Biological Process (BP), the genes were significantly enriched in response to lipopolysaccharide and oxidative stress, the regulation of cell proliferation, and the inflammatory response. Cellular Component (CC) analysis revealed that these targets were primarily localized in the nucleus, cytoplasm, and extracellular space. Regarding Molecular Function (MF), the targets exhibited significant enrichment in identical protein binding, enzyme binding, and transcription factor activity (Figure 1D). Furthermore, KEGG pathway analysis revealed that the core targets were predominantly enriched in landmark signaling pathways, most notably the IL-17 signaling pathway, the TNF signaling pathway, pathways in cancer, and apoptosis (Figure 1E).

### 2.2. Molecular Docking Verification

**Figure 2:**
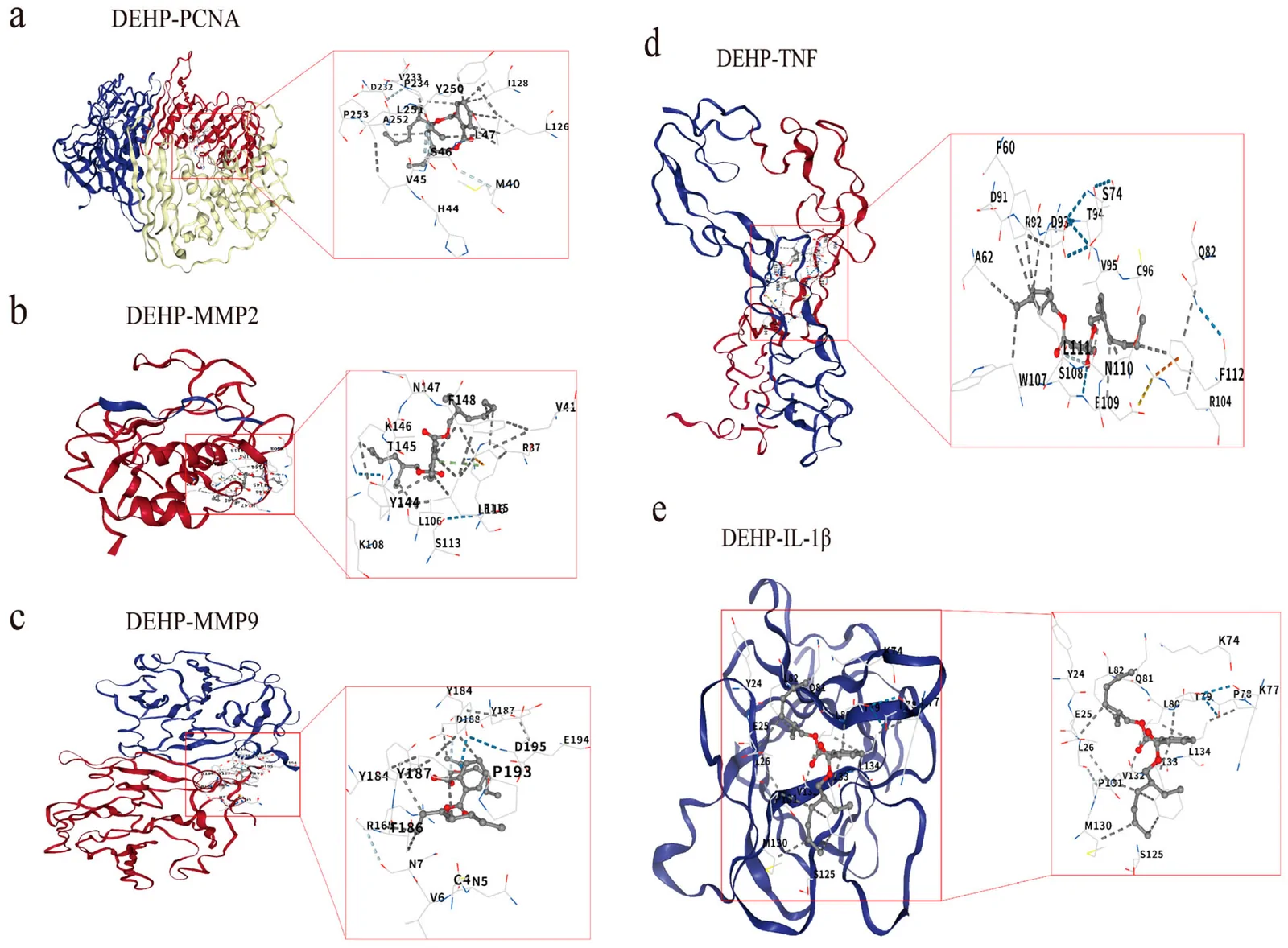

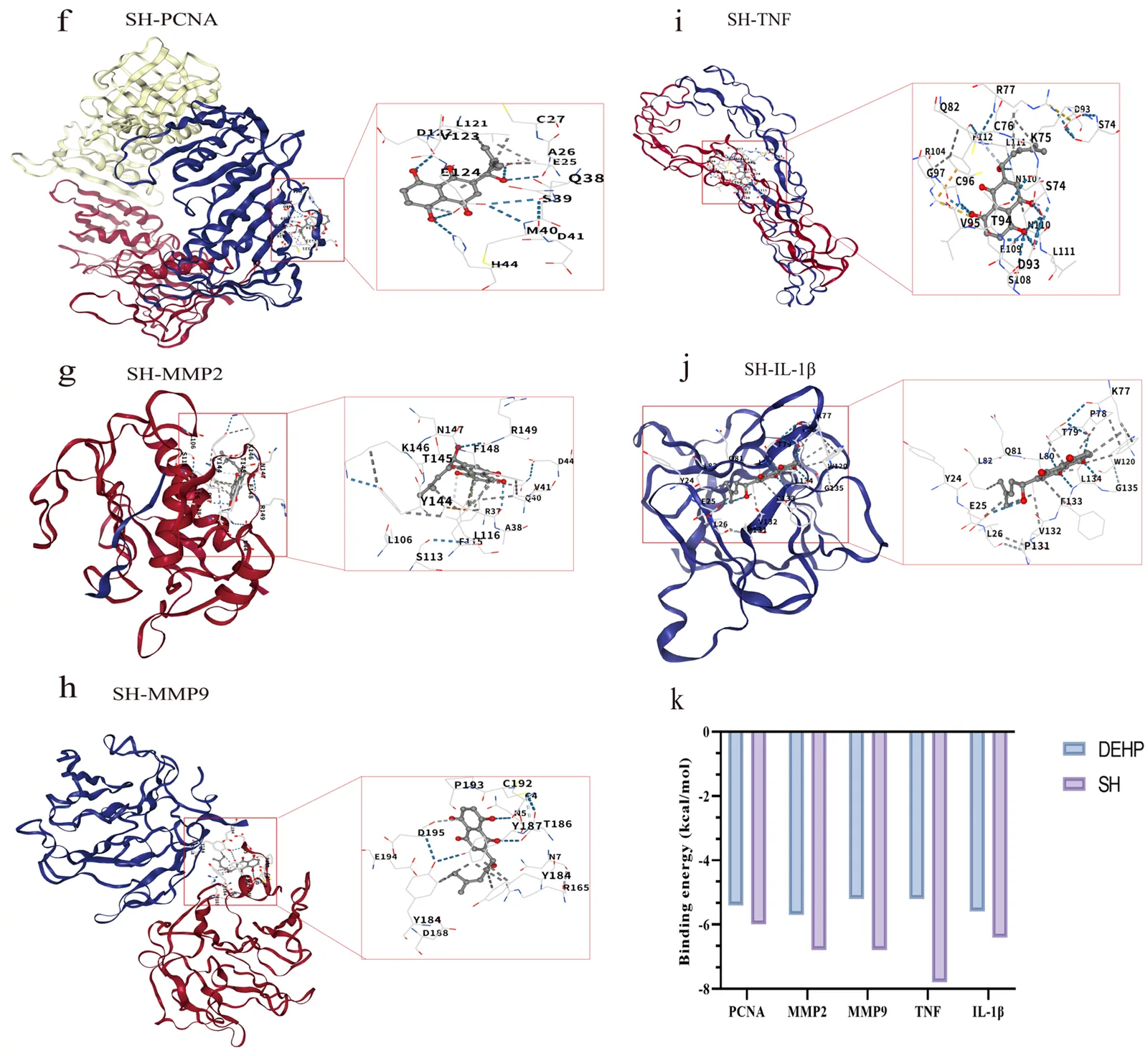
Molecular docking simulations of DEHP and SH with key target proteins. 3D binding poses and detailed interaction interfaces of DEHP with (**a**) PCNA, (**b**) MMP2, (**c**) MMP9, (**d**) TNF, and (**e**) IL-1β. 3D binding poses and detailed interaction interfaces of SH with (**f**) PCNA, (**g**) MMP2, (**h**) MMP9, (**i**) TNF, and (**j**) IL-1Β. The red boxed areas present magnified views of the binding pockets, illustrating the specific interacting amino acid residues and intermolecular interactions (dashed lines represent hydrogen bonds or hydrophobic interactions). (**k**) Comparison of the binding energies (kcal/mol) of DEHP and SH with the respective target proteins. Lower (more negative) binding energy values indicate stronger binding affinities.

To investigate the toxicological mechanism of DEHP and the potential antagonistic effects of SH, molecular docking analysis was performed to detect the binding activity of DEHP and SH with five core target proteins, including PCNA, MMP2, MMP9, TNF, and IL-1β. As shown in Figure 2a–j, the results demonstrated that both DEHP and SH could strongly bind to these targets, with all binding energies consistently falling below the −5.0 kcal/mol threshold. The binding energies for DEHP with the respective targets were −5.4, −5.7, −5.2, −5.2, and −5.6 kcal/mol (Figure 2k). Notably, SH exhibited significantly stronger binding affinities across all identified proteins, yielding lower binding energies of −6.0, −6.8, −6.8, −7.8, and −6.4 kcal/mol, respectively (Figure 2k).

### 2.3. Assessment of Skin Injury Severity via Gross Observation and PASI Scoring

**Figure 3:**
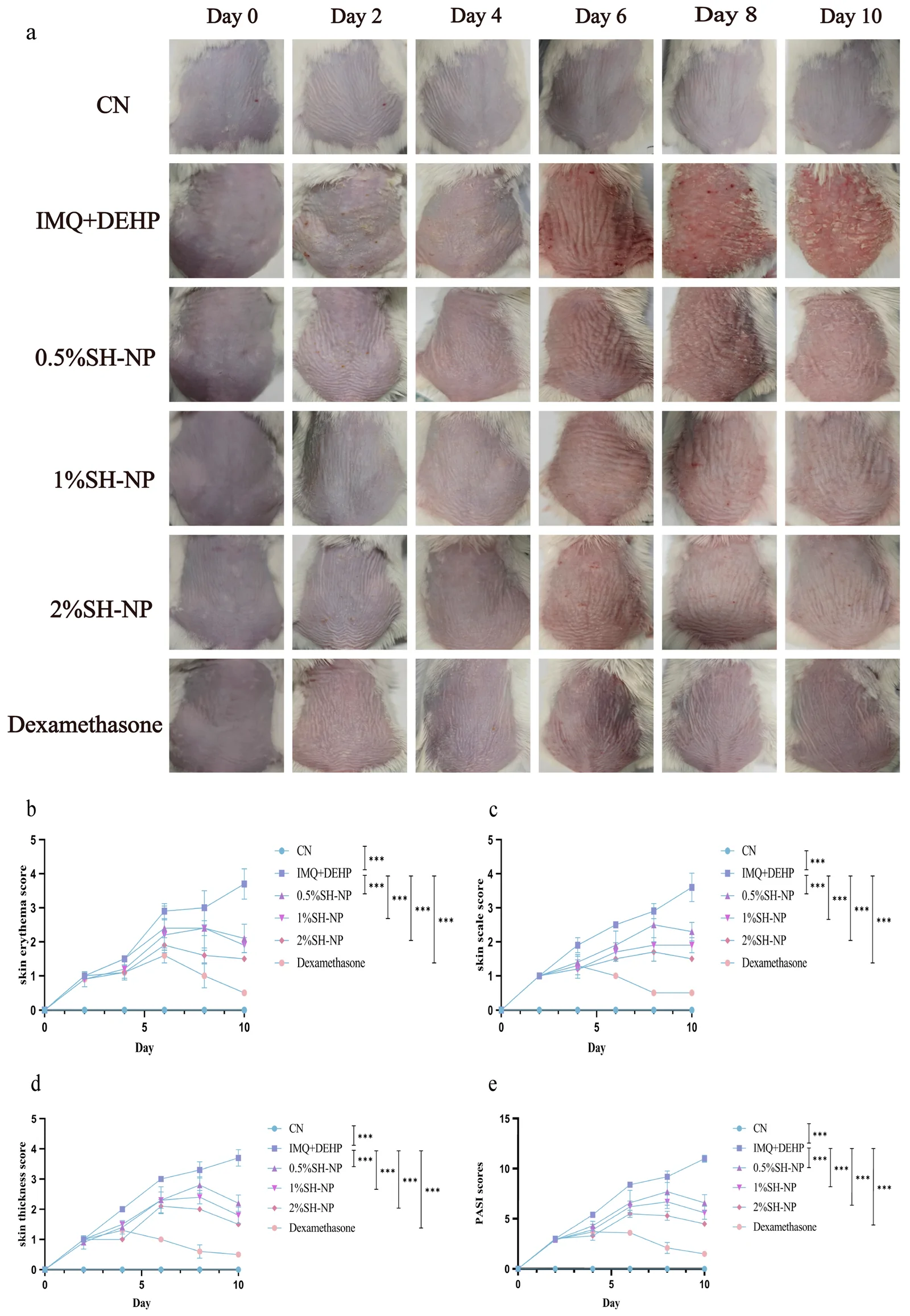
Evaluation of the therapeutic effects of SH-NPs on IMQ- and DEHP-exacerbated psoriasis skin injury in mice. (**a**) Representative photographs of the dorsal skin injury of mice in different treatment groups (CN, IMQ + DEHP, 0.5% SH-NP, 1% SH-NP, 2% SH-NP, and DXM) captured at the indicated time points. (**b**–**e**) Dynamic clinical scoring of the psoriasis-like skin phenotypes over the 10-day experimental period, including (**b**) skin erythema score, (**c**) skin scale score, (**d**) skin thickness score, and (**e**) cumulative Psoriasis Area and Severity Index (PASI) scores. n = 6. Data are expressed as the mean ± SD. *** *p* < 0.001 indicates statistically significant differences compared to the IMQ + DEHP group.

To evaluate the therapeutic efficacy of SH-NPs on psoriasis skin injury exacerbated by the environmental pollutant DEHP, the phenotypic changes in the dorsal skin of mice were monitored over a 10-day experimental period. As illustrated in Figure 3a, the skin of mice in the CN group remained smooth with no signs of inflammation. In contrast, the IMQ + DEHP group exhibited severe psoriasis skin injury, characterized by serious erythema, prominent scaling, and marked skin thickening. These results confirm that DEHP exposure induced significant skin inflammation, consistent with recent toxicological assessments of phthalate esters in inflammatory skin diseases [7]. Notably, as demonstrated in Supplementary Figure S1, compared to the lesions induced by IMQ alone, the IMQ + DEHP co-exposure group exhibited a significantly exacerbated phenotype with more prominent macroscopic scaling and erythema.

Topical administration of SH-NP significantly alleviated the skin injury induced by IMQ and DEHP. Compared to the IMQ + DEHP group, mice treated with 0.5%, 1%, and 2% SH-NPs showed varying degrees of improvement. Crucially, the 2% SH-NP group demonstrated the most pronounced therapeutic effects, with a skin phenotype recovery comparable to that of the DXM group. This potent efficacy mirrors recent advancements in SH-loaded nanoparticle therapies, which have been shown to effectively remodel the skin barrier and suppress systemic inflammatory cascades [33].

To quantify the severity of the injury, scores for erythema, scaling, and thickness were recorded (Figure 3b–d), and the cumulative PASI scores were calculated (Figure 3e). The results indicated that both individual and total PASI scores in the IMQ + DEHP group increased significantly over time, suggesting that DEHP exposure aggravated the skin lesion. Conversely, PASI scores in all SH-NP treatment groups were reduced compared to the model group. The 2% SH-NP treatment effectively alleviated erythema, scaling, and skin thickening, especially in the later stages of the study; its PASI scores were significantly lower than those of the IMQ + DEHP group. These findings demonstrate that SH-NPs effectively mitigate psoriasis skin injury induced by the combination of IMQ and DEHP, with particularly significant efficacy observed at high doses.

### 2.4. Analysis of the Local Immune Cell Infiltration

**Figure 4:**
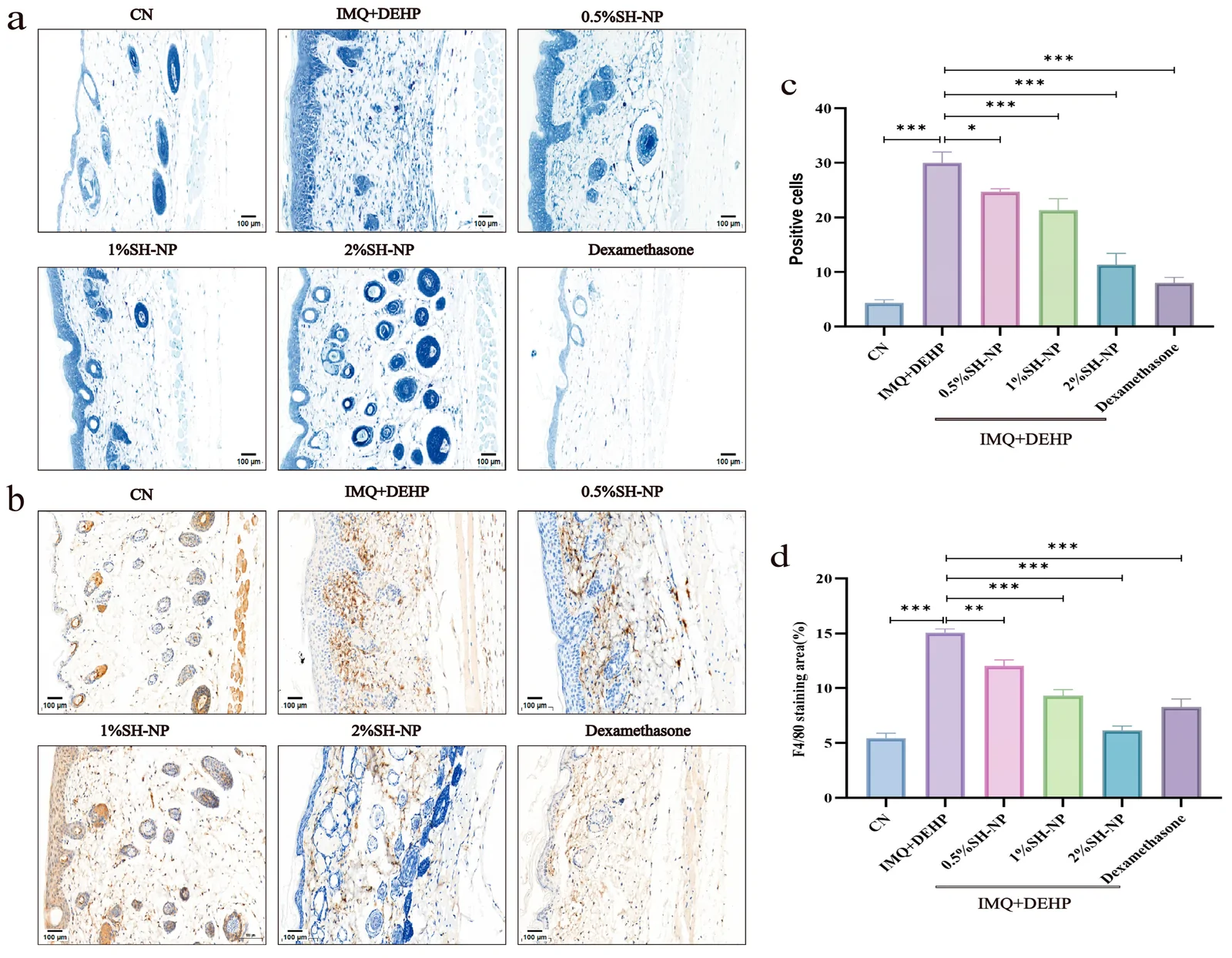
Analysis of mast cell and macrophage infiltration in the dorsal skin of mice. (**a**,**b**) Representative images of (**a**) toluidine blue (TB) staining and (**b**) immunohistochemical staining for F4/80 in skin tissues across different treatment groups. TB stains mast cells, and F4/80 stains macrophages (**c**,**d**). Scale bars = 100 μm. Quantitative analysis of (**c**) mast cells and (**d**) the positive staining area percentages for F4/80, respectively. n = 3. Data are expressed as the mean ± SD. * *p* < 0.05, ** *p* < 0.01, and *** *p* < 0.001 indicate statistically significant differences between the indicated groups.

Since the pathogenesis of psoriasis is deeply intertwined with local inflammatory cascades, dermal immune cell infiltration was assessed (Figure 4) using toluidine blue (TB) staining and F4/80 staining. In the CN group, minimal immune cell presence was observed. Conversely, the IMQ + DEHP group exhibited a robust infiltration and degranulation of mast cells (Figure 4a), alongside a significant accumulation of F4/80-positive macrophages (Figure 4b) in the dermis, indicative of a severe inflammatory microenvironment.

Subsequent administration of SH-NPs dose-dependently mitigated the recruitment of inflammatory cells. The high-concentration group exhibited optimal inhibitory efficacy, restricting the infiltration of mast cells and macrophages (Figure 4c,d). Within this high-dose cohort, quantitative inflammatory cell levels were reduced to an extent comparable to the DXM positive control. Collectively, these results demonstrate that SH-NPs can effectively ameliorate the severe localized inflammatory microenvironment aggravated by DEHP co-exposure.

### 2.5. Evaluation of Skin Histopathological Changes and Epidermal Proliferation

**Figure 5:**
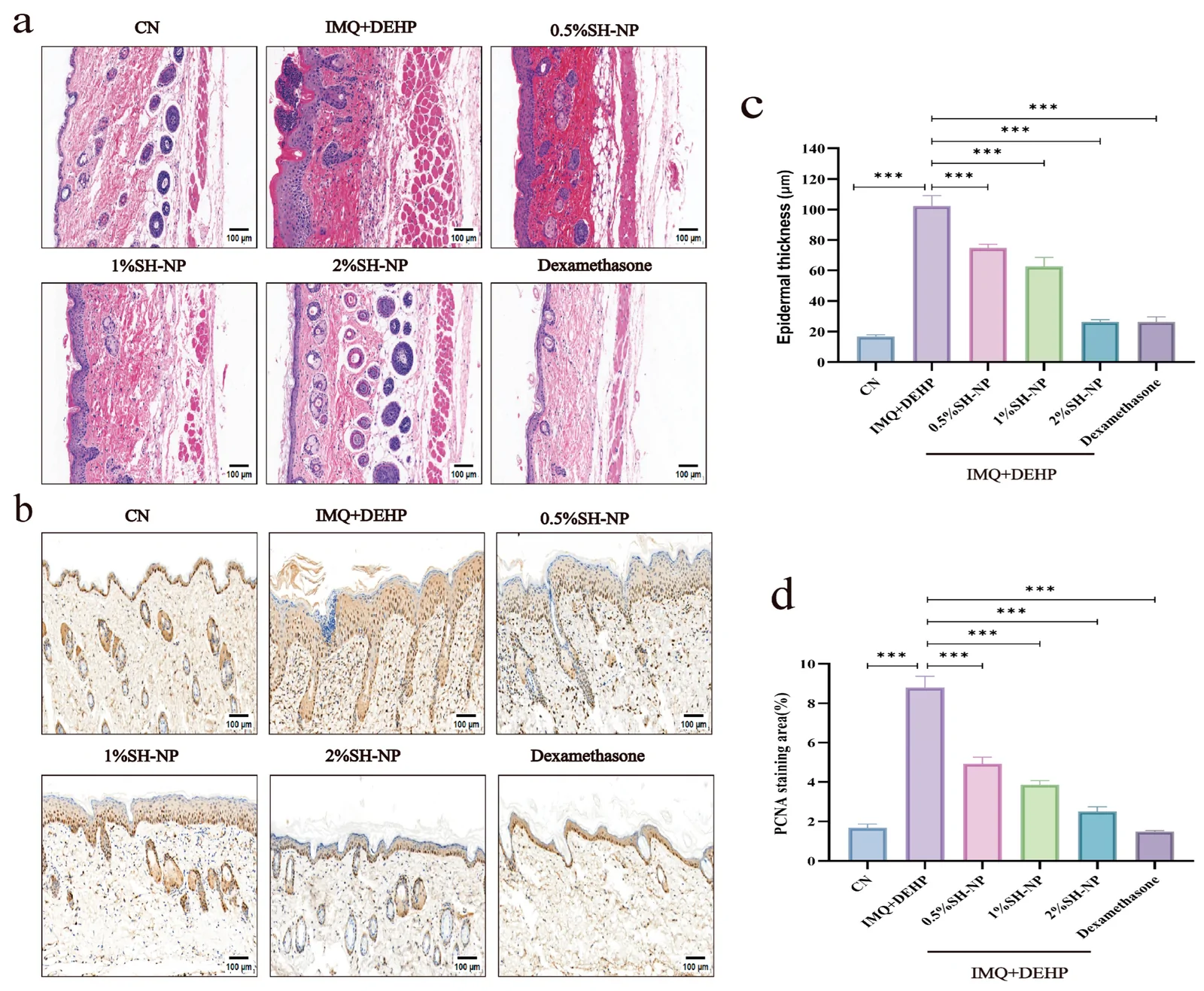
Evaluation of skin histopathological changes and PCNA expression in the dorsal skin of mice. Representative images of (**a**) H&E staining and (**b**) immunohistochemical staining for PCNA in the skin tissues across different treatment groups. The brown color indicates positive staining for the target protein. Scale bars = 100 μm. Quantitative analysis of (**c**) epidermal thickness and (**d**) the positive staining area percentages for PCNA; n = 3. Data are expressed as the mean ± SD. *** *p* < 0.001 indicates statistically significant differences between the indicated groups.

Driven by the aforementioned inflammatory responses, aberrant epidermal hyperproliferation is a hallmark structural consequence. Accordingly, histopathological changes and epithelial cell proliferation were evaluated using H&E and PCNA staining (Figure 5). The CN group exhibited a thin, intact epidermis with normal morphology. In contrast, the IMQ + DEHP group presented with severe histopathological alterations, characterized by pronounced epidermal hyperplasia and excessive cell proliferation, as evidenced by increased epidermal thickness (Figure 5c) and strong PCNA positivity (Figure 5d) in the basal layer. This severe aggravation was further confirmed by comparative H&E staining, which revealed that IMQ + DEHP co-exposure markedly worsened epidermal hyperplasia compared to the IMQ-only phenotype (Supplementary Figure S1).

Therapeutic intervention with SH-NPs attenuated epidermal hyperplasia and downregulated cellular proliferation. Notably, the high-dose SH-NP treatment yielded optimal therapeutic efficacy. It significantly suppressed aberrant PCNA expression (Figure 5d), reduced epidermal thickness (Figure 5c), and restored the epidermal architecture to a state closely resembling the normal tissue profile.

### 2.6. Extracellular Matrix Remodeling and Matrix Metalloproteinase Expression

**Figure 6:**
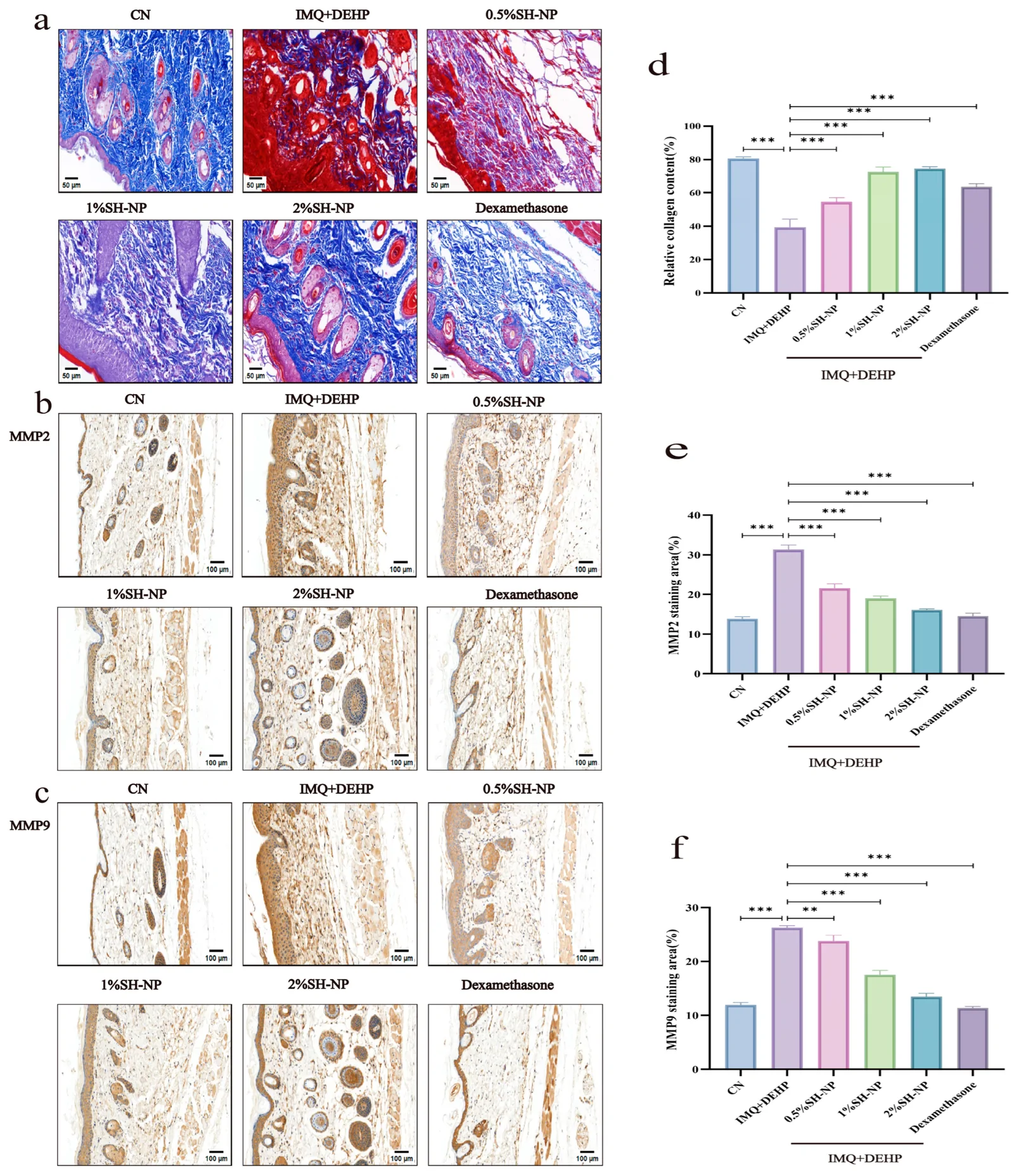
Extracellular matrix remodeling and expression of MMP2 and MMP9 in the skin injury of mice. Representative images showing (**a**) Masson’s trichrome staining (blue color indicates collagen fibers), and the immunohistochemical expression levels of (**b**) MMP2 and (**c**) MMP9 in the skin tissues across different treatment groups. The brown color indicates positive staining for the target proteins. Scale bars = 50 μm in (**a**) and 100 μm in (**b**,**c**). Quantitative analysis of (**d**) relative collagen content, and the positive staining area percentages for (**e**) MMP2 and (**f**) MMP9. n = 3. Data are expressed as the mean ± SD. ** *p* < 0.01, *** *p* < 0.001 indicates statistically significant differences between the indicated groups.

Furthermore, the integrity of the dermal extracellular matrix (ECM) and MMP2/9 was investigated (Figure 6). The CN group displayed dense, well-organized collagen bundles with basal expression levels of MMP2/9. Following IMQ + DEHP exposure, a marked upregulation of MMP2/9 (Figure 6b,c) was observed, concurrent with severe fragmentation and degradation of collagen (Figure 6a).

Administration of 0.5% and 1% SH-NPs moderately downregulated MMP2/9 expression, conferring partial protection against collagen depletion. Crucially, the 2% SH-NP treatment yielded the most potent protective effect. By significantly inhibiting the overexpression of MMP2 and MMP9 (Figure 6e,f), the 2% formulation successfully preserved the relative collagen content and structural organization of the ECM (Figure 6d), demonstrating superior efficacy in preventing tissue remodeling and structural degradation.

### 2.7. Assessment of Spleen Index

**Figure 7:**
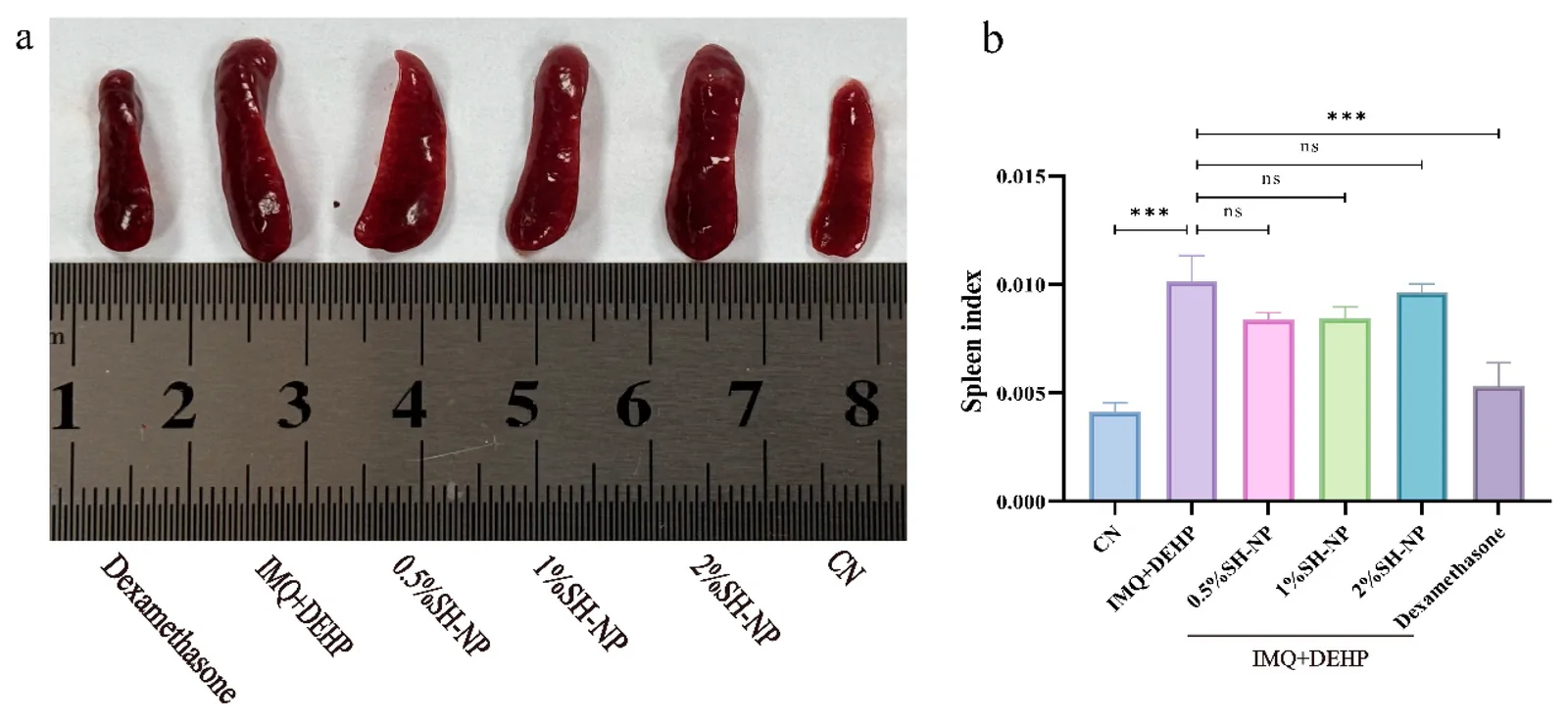
Evaluation of systemic immune response via macroscopic spleen observation and spleen index. (**a**) Representative macroscopic images of spleens isolated from mice in the different treatment groups at the end of the experiment. (**b**) Spleen index of mice across the different groups. n = 6. Data are expressed as the mean ± SD. *** *p* < 0.001 indicates statistically significant differences between the indicated groups; ns indicates no significant difference.

Splenomegaly serves as a prominent systemic indicator of immune hyperactivation in the psoriasis model. To evaluate the impact of topically applied SH-NPs on this systemic inflammatory response, macroscopic changes in the spleens and spleen indices were evaluated (Figure 7). As depicted in Figure 7a, spleens isolated from the IMQ + DEHP group were visibly enlarged and engorged compared to the normal-sized spleens of the CN group. Quantitative analysis (Figure 7b) confirmed this, demonstrating a significantly elevated spleen index in the IMQ + DEHP group, which indicates robust systemic immune activation driven by the combined exposure. Following the topical administration of SH-NPs, although the spleen indices exhibited a slight downward trend, statistical analysis revealed no significant differences across all SH-NP treatment groups compared to the model group. In contrast, treatment with the positive control drug, DXM, significantly reduced the spleen index, reflecting its typical systemic immunosuppressive effects. These findings suggest topically applied SH-NPs are predominantly confined to the cutaneous inflammatory microenvironment, effectively suppressing local skin injury. The restoration of the spleen index primarily reflects relief from the overflow of systemic inflammation. Furthermore, by mitigating this systemic inflammatory burden without inducing the lymphoid atrophy typically associated with traditional corticosteroid therapies like DXM, SH-NPs may not induce systemic immunosuppression.

### 2.8. Analysis of mRNA Expression Levels of Pro-Inflammatory Cytokines in Skin Tissue

**Figure 8:**
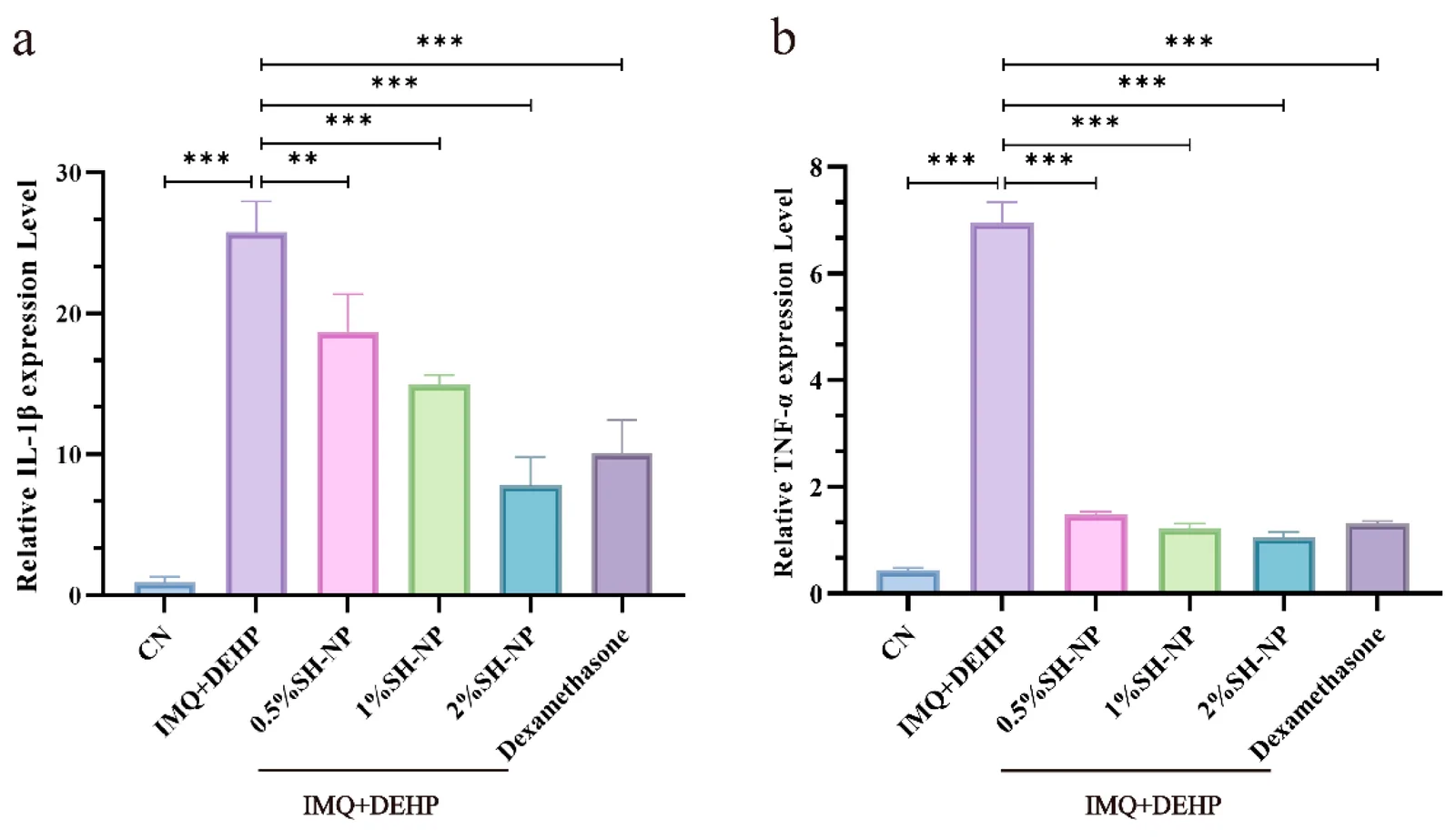
Relative mRNA expression levels of inflammatory cytokines in the skin injury of mice. qRT-PCR analysis of (**a**) IL-1β and (**b**) TNF-α expression in the skin tissues of different treatment groups. n = 3. Data are expressed as the mean ± SD. ** *p* < 0.01 and *** *p* < 0.001 indicate statistically significant differences between the indicated groups.

The relative mRNA expression levels of key pro-inflammatory cytokines, IL-1β and TNF-α, in the dorsal skin of injured mice were detected using qRT-PCR. As shown in Figure 8a,b, compared with the CN group, the relative mRNA expression of IL-1β and TNF-α in the skin tissues was significantly upregulated in the IMQ + DEHP group, indicating the induction of a robust inflammatory response. Following treatment with different concentrations of SH-NPs, the expression levels of both inflammatory cytokines showed a significant dose-dependent decrease compared to the model group. These results demonstrate that SH-NP possesses excellent anti-inflammatory efficacy and can effectively mitigate the inflammatory damage induced by IMQ and DEHP.

### 2.9. Inhibitory Effect of SH-NPs on the Activation of the p38 MAPK Signaling Pathway

**Figure 9:**
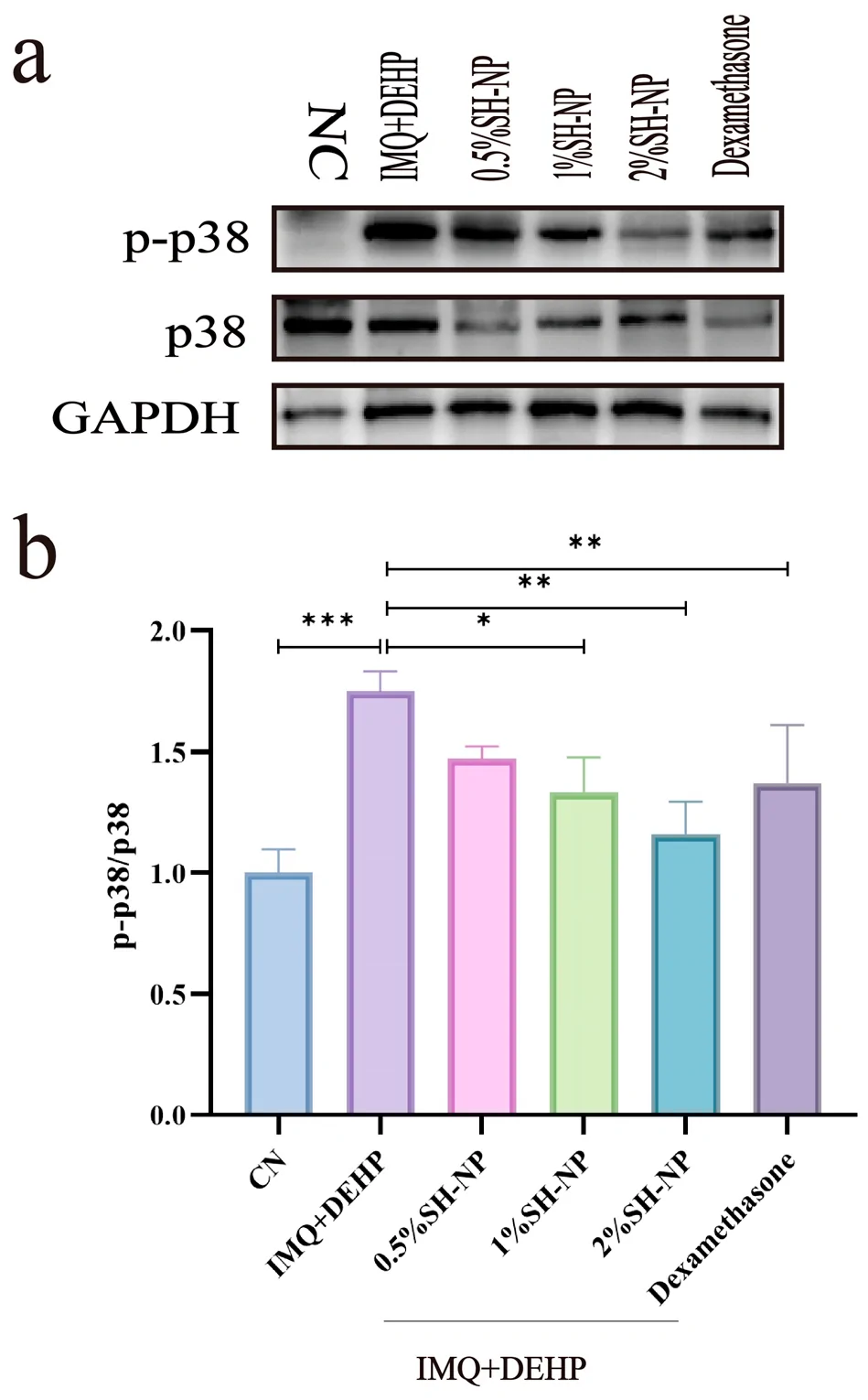
Inhibitory effect of SH-NPs on p38 MAPK phosphorylation in skin injury in mice. (**a**) Representative Western blot images showing the protein expression levels of phosphorylated p38 (p-p38), total p38, and GAPDH across different treatment groups. GAPDH was used as an internal loading control. (**b**) Quantitative densitometric analysis of the relative p-p38/p38 ratio. n = 3. Data are expressed as the mean ± SD. * *p* < 0.05, ** *p* < 0.01, and *** *p* < 0.001 indicate statistically significant differences between the indicated groups.

Western blot analysis was performed to evaluate the impact of SH-NPs on the activation of p38 MAPK. The results (Figure 9) revealed that, compared to the normal CN group, the expression levels of phosphorylated p38 (p-p38) and the ratio of p-p38/p38 were significantly elevated in the IMQ + DEHP group. SH-NP treatment substantially suppressed the phosphorylation of p38 and the p-p38/p38 ratio in a dose-dependent manner, achieving an efficacy comparable to that of the positive control, DXM. These findings indicate that the therapeutic effects of SH-NPs on damaged skin tissues may be mediated through the regulation of the p38 MAPK signaling pathway.

## 3. Discussion

While previous studies reported the anti-psoriatic potential of shikonin (SH), traditional applications predominantly utilized free SH monomers, which are limited by poor topical bioavailability. Nano-encapsulation has emerged to overcome these physicochemical barriers. Consistent with the existing literature demonstrating that nanoparticle delivery significantly enhances the anti-inflammatory efficacy of hydrophobic compounds [34,35], our study rationally employed SH-NPs to ensure optimal therapeutic outcomes. Furthermore, this research advances the field by shifting from classic experimental setups to an epidemiologically relevant context. Instead of an isolated IMQ model, we used a bioinformatics-guided approach to evaluate SH-NPs in a DEHP-exacerbated psoriasis model to better reflect real-world environmental triggers.

To explore the mechanisms underlying DEHP-exacerbated psoriasis skin damage and to evaluate the therapeutic efficacy of SH-NPs, this study utilized a combined “prediction-to-validation” approach. First, results from network toxicology and network pharmacology showed a clear link between inflammation, cell growth, and tissue remodeling, with TNF-α, IL-1β, PCNA, and MMP2/9 emerging as the main shared targets. Molecular docking showed that DEHP and SH bind strongly to these proteins, which suggests that SH can directly block DEHP-exacerbated harmful signals to exert skin-protective effects.

Secondly, a mouse model of DEHP and IMQ co-exposure was established to validate this “toxicant–disease–drug” axis. Our in vivo results clearly confirmed the dermatological toxicity of DEHP exposure, evidenced by severe psoriasis injury, elevated PASI scores, and significant epidermal thickening. Furthermore, we demonstrated that the topical application of SH-NPs significantly ameliorated these psoriasis injuries, exhibiting efficacy comparable to that of DXM. Importantly, while traditional corticosteroids like DXM drastically reduced the spleen index and indicated severe lymphoid atrophy, SH-NPs maintained macroscopic organ homeostasis. The normalized spleen index in the SH-NP groups appears to reflect an attenuation of the secondary systemic stress triggered by the severe cutaneous lesions. These observations imply that the anti-inflammatory action of SH-NPs is potentially confined to the localized cutaneous microenvironment. Consequently, this targeted topical action may help avoid the severe systemic immunotoxic side effects commonly associated with traditional steroids. Our previous study showed that SH-NPs within the same concentration range exhibit no observable toxicity to the heart, lungs, liver, or kidneys [36]. In summary, the present study’s combination of exposure context, formulation, and multi-domain readouts firmly proved both the dermal toxicity of DEHP and the potent, localized protective effects of SH-NPs, providing a solid foundation for their potential clinical application in environmentally exacerbated psoriasis.

As mentioned above, immune cell infiltration and inflammatory factor production play an important role in the progression of psoriasis. Through genetic analysis, Zou et al. demonstrated that the genes targeted by DEHP are clustered in core psoriasis pathways, such as TNF and MAPK [7]; however, they did not validate these results. Our bioinformatic analysis showed that SH targets multiple inflammation-related pathways, specifically the TNF signaling pathway and MAPK-related pathways, to counter DEHP toxicity. Consistent with this, our in vivo results clearly proved an increased accumulation of macrophages and mast cells infiltrating the skin tissue of DEHP + IMQ-exposed mice. This cellular infiltration was accompanied by elevated levels of TNF-α and IL-1β, as well as the overactivation of the p38 MAPK signaling pathway. In contrast, SH-NP treatment significantly decreased the infiltration of these immune cells, suppressed p38 activation, and downregulated TNF-α and IL-1β mRNA levels. These results demonstrate that SH-NPs can effectively mitigate DEHP-driven local inflammation by modulating the p38 MAPK pathway. Our results align with the recent literature indicating that various natural plant-derived compounds can alleviate psoriasis by suppressing MAPK signaling cascades [14,37]. For instance, Bai et al. demonstrated that eupatilin, a naturally occurring flavone, significantly inhibited keratinocyte hyperproliferation and ameliorated imiquimod-induced psoriasis skin injury in mice by blocking the p38 MAPK pathway [14]. Similarly, Fu et al. reported that 4′-O-β-D-glucosyl-5-O-methylvisamminol (4GMV), an active chromone extracted from traditional medicinal plants, exerted potent anti-psoriatic effects by downregulating MAPK, thereby reducing inflammatory cell infiltration and the release of pro-inflammatory cytokines [37]. These findings further confirm the therapeutic value of targeting the p38 pathway with natural agents.

In addition to inflammation, epidermal hyperproliferation is a hallmark pathological feature of psoriasis. PCNA is a key biological marker reflecting DNA synthesis and cell proliferation. Our bioinformatics analysis revealed that many targets shared by DEHP and SH are enriched in pathways related to cancer and apoptosis, which biologically translates to the regulation of abnormal cell division and survival seen in psoriatic hyperplasia. Linking this to our animal model, in vivo tests showed that DEHP + IMQ exposure drastically increased PCNA expression, directly correlating with the observed enhancement of epithelial thickness. Following SH-NP treatment, PCNA levels were significantly downregulated, confirming the formulation’s ability to halt excessive keratinocyte proliferation and resolve epidermal thickening. This anti-proliferative effect is consistent with other studies highlighting the ability of targeted natural therapies to normalize epidermal turnover in psoriatic models [38,39]. For instance, Le et al. demonstrated that Taodan granules, a botanical formulation, robustly suppressed epidermal hyperplasia and significantly reduced the number of PCNA-positive cells in the epidermis of IMQ-induced mice [38]. Furthermore, a recent comprehensive review by Lee et al. emphasizes that inhibiting aberrant cell division pathways and promoting apoptosis in hyperproliferative keratinocytes serves as a shared, fundamental mechanism for many successful natural-product-derived anti-psoriatic agents [39]. So, our results proved that disruption of the abnormal proliferation cycle may be one of the important mechanisms by which SH-NPs exert their protective effects in psoriasis.

Simultaneously, the maintenance of the dermal barrier is heavily dependent on the balance of extracellular matrix degradation. MMPs, particularly MMP2 and MMP9, are the primary enzymes responsible for tissue remodeling. Overactive MMPs accelerate the breakdown of the dermal matrix, which not only disrupts the skin barrier but also physically facilitates the deeper infiltration of inflammatory cells into the skin [40]. Our study found that SH-NPs strongly suppressed the elevated levels of MMP2 and MMP9 induced by DEHP. This inhibitory effect largely explains the parallel reduction in macrophage infiltration and the restoration of organized collagen deposition observed in our histological evaluations. Interestingly, while conventional psoriasis treatments focus predominantly on immune cytokines, our results highlight a distinct, supplementary benefit of SH-NPs in directly regulating tissue-remodeling enzymes. Similar dual-action capabilities—simultaneously suppressing inflammation and actively promoting skin barrier repair—have recently been reported for other emerging nano-formulations delivering natural active agents [41,42].

Although this study successfully demonstrated the toxic targets of DEHP and the therapeutic value of SH-NPs, several limitations remain. First, our choice of the co-exposure protocol mainly relied on prior controlled studies [9,10] that had already examined the relevant component groups. Consistent with these literature reports, our preliminary experiments also confirmed that IMQ + DEHP co-exposure significantly exacerbated psoriasis (Supplementary Figure S1). Therefore, we did not include a separate IMQ-only group in our experiments because the therapeutic effects of shikonin on IMQ-induced psoriasis have been reported. As previous studies have proved that DEHP alone cannot induce psoriasis, we also did not include a separate DEHP-only group. Nevertheless, we still believe that the inclusion of these necessary control groups would render the results more complete and compelling. Similarly, formulation-centric controls, such as free shikonin and blank nanoparticles, were excluded, limiting our ability to quantify the specific transdermal advantages of the nanocarrier versus the free drug [43]. Second, the precise molecular mechanisms remain insufficiently explored. Although our network directed our focus to p38 MAPK and macrophage/mast cell infiltration, the absence of JNK/ERK pathway analysis, IL-6 detection, and broader T-cell profiling represents limitations. Furthermore, while the IL-17A axis is critical in DEHP-related psoriasis [10], it was not investigated here and must be addressed in subsequent research. Third, the lack of specific p38 MAPK pharmacological inhibitors means our current in vivo mechanistic evidence remains correlative. Fourth, while spleen index data indicated an absence of macroscopic splenomegaly, comprehensive serum cytokine profiling and organ histology are strictly required to conclusively confirm the avoidance of systemic immunosuppression. Finally, while the DEHP + IMQ model mimics pollutant-associated conditions, species differences necessitate broader disease models to validate clinical translatability, and future investigations must systematically evaluate the transdermal absorption kinetics and long-term toxicological safety profile of these nanoparticles.

In summary, this study integrated network toxicology, network pharmacology, molecular docking, and in vivo experiments to identify the pathogenic targets of DEHP and validate the comprehensive protective effects of SH. We demonstrated that SH-NPs effectively ameliorate DEHP-associated psoriasis skin injury by inhibiting the p38 MAPK inflammatory pathway, arresting abnormal cellular proliferation, and normalizing MMP-driven tissue remodeling. Our findings firmly indicate that SH-NPs possess potent, localized anti-inflammatory and barrier-repairing properties, providing robust preclinical evidence for the development of targeted nanotherapeutics against environmentally associated dermatological diseases.

## 4. Materials and Methods

### 4.1. Network Toxicology, Network Pharmacology and Molecular Docking

#### 4.1.1. Screening of DEHP-Related Toxicity Targets

The canonical SMILES string of DEHP was obtained from the PubChem database (https://pubchem.ncbi.nlm.nih.gov/), accessed on 31 July 2026 [44]. This SMILES string was then submitted to SwissTargetPrediction (http://www.swisstargetprediction.ch/) accessed on 31 July 2026 to predict potential protein targets based on structural similarity, with the organism restricted to “*Homo sapiens*” and a probability threshold of >0 [45]. Concurrently, the Comparative Toxicogenomics Database (CTD, http://ctdbase.org/) accessed on 31 July 2026 was screened for genes associated with DEHP using the keyword “di (2-ethylhexyl) phthalate or DEHP” [46]. To ensure the reliability of the evidence and filter out low-confidence associations, targets with an Interaction Count ≥ 5 were retained. Experimentally validated targets were retrieved from the ChEMBL database (https://www.ebi.ac.uk/chembl/) accessed on 31 July 2026 to supplement the dataset with verified pharmacological interaction data [47]. Finally, all retrieved targets were standardized to official gene symbols using the UniProt Knowledgebase (https://www.uniprot.org/) accessed on 31 July 2026, and duplicates were removed to establish the final DEHP toxicity target set.

#### 4.1.2. Acquisition of Disease Targets Related to Psoriasis

Psoriasis targets were gathered by integrating data from GeneCards (https://www.genecards.org/) accessed on 31 July 2026 [48], the Online Mendelian Inheritance in Man (OMIM, https://omim.org/) accessed on 31 July 2026 [49], and DrugBank (https://go.drugbank.com/) accessed on 31 July 2026 [50]. To ensure data reliability, genes from GeneCards were filtered by a Relevance Score ≥ 1, while OMIM and DrugBank were utilized to capture core genetic drivers and validated therapeutic targets, respectively. The datasets were merged, deduplicated, and standardized through the UniProt Knowledgebase to form a collection of psoriasis disease targets.

#### 4.1.3. Targets of Shikonin

To identify the potential therapeutic targets of SH, a combined approach integrating pharmacophore mapping, similarity prediction, and text mining was applied. The 3D structure of SH was submitted to the PharmMapper server (http://www.lilab-ecust.cn/pharmmapper/) accessed on 31 July 2026 [51] for potential target identification via an inverse docking approach. To ensure high-confidence predictions, only targets with a Norm Fit score > 0.9 were retained. Then, SwissTargetPrediction was employed to predict targets based on the 2D chemical structure of SH under the “*Homo sapiens*” setting (Probability > 0). The CTD database was queried to supplement the dataset with literature-curated interactions involving SH. The candidate targets from these three sources were pooled, and the final SH target set was established after removing duplicates and standardizing gene symbols via the UniProt Knowledgebase.

#### 4.1.4. Construction of the “DEHP–Psoriasis–Shikonin” Network

To elucidate the potential targets by which DEHP exacerbates psoriatic conditions, the predicted DEHP-related targets were first cross-referenced with psoriasis-associated genes using the jvenn tool (http://bioinfo.genotoul.fr/jvenn) accessed on 31 July 2026. This initial intersection aimed to identify the pathological genes responsible for DEHP-exacerbated aggravation of psoriasis. Subsequently, to reveal the precise pharmacological mechanisms of SH against this specific aggravated state, the SH-related targets were mapped onto the aforementioned intersection. This tripartite convergence was performed to screen and isolate the core candidate targets, thereby representing the key molecular nodes through which SH exerts its therapeutic efficacy on DEHP-aggravated psoriasis.

#### 4.1.5. PPI Network Construction and Topological Analysis

Core candidate targets were imported into the STRING database (https://cn.string-db.org/) accessed on 31 July 2026 [52] to construct a protein–protein interaction (PPI) network (confidence score > 0.7) and visualized using Cytoscape (version 3.10.3). The “Network Analyzer” tool was utilized to analyze the topology of the PPI network. With “Degree”, “Betweenness”, and “Closeness” as references, key targets were screened in combination with literature reports. To identify the most critical functional nodes in the complex network, the cytoHubba plugin was used, while the Maximal Clique Centrality (MCC) algorithm was applied to rank the nodes. The top-ranking genes with the highest MCC scores were identified as key hub genes responsible for core biological functions.

#### 4.1.6. Enrichment Analysis

The obtained core candidate targets were imported into the DAVID database (https://david.ncifcrf.gov/) accessed on 31 July 2026 for Gene Ontology (GO) annotation and Kyoto Encyclopedia of Genes and Genomes (KEGG) pathway enrichment analysis. The core candidate targets were uploaded to the server, with identifiers set to “OFFICIAL_GENE_SYMBOL”and the species selected as “*Homo sapiens*”. Bioinformatics tools were used to visualize the top-ranked entries.

#### 4.1.7. Molecular Docking

Molecular docking was performed to investigate the binding interactions of DEHP and SH with key hub targets using the CB-Dock2 web server (http://cadd.labshare.cn/cb-dock2/) accessed on 31 July 2026 [53]. The CurPocket algorithm, a curvature-based cavity detection method, was employed to automatically identify potential binding pockets on the protein surfaces, followed by docking simulations using AutoDock Vina (version 1.5.7). The 3D structures of the hub proteins were retrieved from the Protein Data Bank (PDB, https://www.rcsb.org/) accessed on 31 July 2026 [54], while the chemical structures of SH and DEHP were obtained from PubChem. Among the top-ranked cavities, the conformation with the lowest Vina score was selected as the optimal binding pose. A Vina score of less than −5.0 kcal/mol was defined as the threshold for stable binding activity.

### 4.2. In Vivo Validation

#### 4.2.1. Experimental Animals

The animal study protocol was approved by the Institutional Animal Care and Use Committee (IACUC) of Guangdong Pharmaceutical University (Approval No. gdpulacspf2022948, approved date: 24 October 2025). All experimental procedures were conducted in strict accordance with the Guidelines for the Care and Use of Laboratory Animals of the National Institutes of Health.

A total of 36 male BALB/c mice (aged 6–8 weeks; weighing 18 ± 2 g; SPF grade) were purchased from Guangzhou Ruige Biotechnology Co., Ltd. The mice were housed in a temperature-controlled environment (23 ± 2 °C) with a humidity of 45–55% under a 12 h light/12 h dark cycle. All animals had ad libitum access to standard chow and water. Prior to the experiment, the mice were acclimatized to the laboratory conditions for 7 days.

#### 4.2.2. Experimental Design

One day prior to model induction, the dorsal hair of all mice was removed using a non-irritating depilatory cream to expose a skin area of approximately 2 cm × 4 cm. Following the acclimatization period, thirty-six male BALB/c mice were randomly assigned into six experimental groups (n = 6 per group) and subjected to a 10-day consecutive treatment protocol. The healthy control group received daily subcutaneous injections of normal saline without model induction. For the remaining five groups, a psoriasis-like model was established daily in the morning via a subcutaneous injection of DEHP (40 μg/kg/day, Sigma-Aldrich, St. Louis, MO, USA; Cat. No. D201154), combined with topical application of 62.5 mg of 5% IMQ cream (Hubei Keyi Pharmaceutical Co., Ltd., Wuhan, China; Lot No. 250201) to the exposed dorsal skin [9,55].

Concurrently, these induced mice were treated with topical applications of their respective therapeutic interventions. The model group received a topical application of the blank vehicle. The SH-NP treatment groups were treated with topical applications of SH-NP suspensions at different concentrations every afternoon. The SH-NPs used in this study were synthesized and purified according to our previous reports, with their identity and purity verified prior to use [36]. As strictly characterized in that report, the standardized formulation documented a reliable hydrodynamic diameter of approximately 209 nm, a polydispersity index (PDI) of 0.064, a zeta potential of −17.9 mV, and an encapsulation efficiency of 88%. It should be noted that SH-NPs were utilized primarily as a delivery tool to overcome the severe hydrophobicity and poor topical bioavailability of raw shikonin. To adhere to the 3R principles of animal welfare and according to prior reports [43], formulation-centric controls (i.e., free shikonin and blank nanoparticles) were omitted, directing the in vivo cohort entirely toward validating the dose-dependent mechanisms of SH-NPs within the DEHP-exacerbated network. The positive control group was treated with a topical application of 2 mg/kg dexamethasone (DXM, China Resources Sanjiu Medical & Pharmaceutical Co., Ltd., Shenzhen, China) in the afternoon [56]. Throughout the experimental period, the body weight and health status of the mice were monitored daily. At the study’s endpoint, all animals were euthanized via CO_2_ inhalation, and biological samples were collected for further analysis.

#### 4.2.3. Observation and Evaluation of Skin Injury

Throughout the experimental period, macroscopic changes in the dorsal skin injuries of the mice were monitored and photographed at designated time points using a digital camera. The severity of the psoriasis skin injury was evaluated using a clinical scoring system based on the Psoriasis Area and Severity Index (PASI). Specifically, the severity of erythema, scaling, and skin thickening was independently scored on a scale from 0 to 4 (0 = none; 1 = mild; 2 = moderate; 3 = severe; 4 = very severe). A cumulative score (ranging from 0 to 12) was then calculated to represent the overall severity of the skin injury. To minimize subjective bias, all macroscopic evaluations and clinical scoring were performed by two independent investigators who were strictly blinded to the experimental group assignments.

#### 4.2.4. Histological and Histochemical Analysis

The mouse dorsal skin tissues were fixed in 4% paraformaldehyde for 24 h, followed by dehydration in a graded series of ethanol, clearing in xylene, and embedding in paraffin. The paraffin-embedded tissues were then sectioned into 5 μm-thick slices using a microtome. For morphological evaluation, the skin sections were stained with hematoxylin and eosin (H&E) to observe general pathological changes. To assess collagen deposition, Masson′s trichrome staining was performed according to the manufacturer′s instructions. For the detection and quantification of mast cell infiltration, the sections were stained with 0.1% toluidine blue. All histological slides were observed and captured using an optical microscope. For quantitative analysis, five randomly selected, tissue-specific high-power fields per section were evaluated (the complete epidermis for thickness and the dermis for collagen and mast cells). To ensure objectivity, quantification was performed using ImageJ software (version 1.54g) with uniform thresholding criteria by two independent investigators strictly blinded to the group assignments. Measurements from the five technical fields were averaged to yield a single data point per animal, ensuring that the n value strictly represents independent biological replicates (individual mice) rather than technical fields [55].

#### 4.2.5. Immunohistochemistry (IHC) Analysis

Paraffin-embedded skin tissue sections were deparaffinized in xylene and rehydrated through a graded series of ethanol. Heat-induced antigen retrieval was performed in citrate buffer (pH 6.0) under high pressure for 2 min, followed by natural cooling to room temperature. To quench endogenous peroxidase activity, the sections were incubated in 3% H_2_O_2_ for 20 min. Non-specific binding was blocked with 10% normal goat serum (Pinuofei Biotech, Wuhan, China) at 37 °C for 30 min. Subsequently, the sections were incubated overnight at 4 °C with the following primary antibodies: anti-PCNA (1:1000, Proteintech, Rosemont, UK), anti-MMP9 (1:1000, Servicebio, Wuhan, China), anti-MMP2 (1:1000, Servicebio), and anti-F4/80 (1:500, ABCAM, Cambridge, UK). After being washed with PBS three times, the sections were incubated with an HRP-conjugated goat anti-rabbit secondary antibody (Pinuofei Biotech) for 1 h at 37 °C. Immunoreactivity was visualized using a 3,3′-diaminobenzidine (DAB) chromogen kit. Then, the sections were counterstained with hematoxylin for 3 min, differentiated with 0.5% hydrochloric acid–ethanol, dehydrated, and mounted with neutral resin for microscopic examination. Quantitative analysis of positive staining areas was performed using ImageJ software, applying uniform thresholding criteria across all samples. To prevent selection bias, evaluations were independently conducted by two blinded investigators across five randomly selected, tissue-specific high-power fields per section (the epidermis for PCNA, and the dermis for MMPs and F4/80). The data from these five technical fields were averaged to generate a single representative value per animal, definitively confirming that the n value represents independent biological replicates [57].

#### 4.2.6. Quantitative Real-Time PCR (qRT-PCR)

The expression levels of inflammatory factors (TNF-α and IL-1β) were measured by qRT-PCR. Briefly, total RNA was extracted from skin tissues using an RNA extraction kit (Albatross, Guangzhou, China) according to the manufacturer’s instructions. The concentration and purity of RNA were determined using a Nano-300 instrument (Allsheng, Hangzhou, China). Complementary DNA (cDNA) was synthesized using a commercial reverse transcription kit, according to the manufacturer’s instructions (Albatross, Guangzhou, China). qRT-PCR was performed using a SYBR Green kit (Albatross, Guangzhou, China) on a QuantStudio 6 Pro. The expression levels of TNF-α and IL-1β were normalized to the housekeeping gene β-actin and calculated using the 2^(−ΔΔCT)^ method. The primer sequences used in this study are listed in Table 1.

#### 4.2.7. Western Blot Analysis

Total proteins were extracted from the whole full-thickness skin tissue (epidermis and dermis combined) of the lesional area using RIPA lysis buffer containing protease and phosphatase inhibitors. The protein concentrations were determined using a BCA Protein Assay Kit (Cat. No. WB6501, Xinsaimei, Suzhou, China). Equal amounts of protein extracts were separated by sodium dodecyl sulfate–polyacrylamide gel electrophoresis (SDS-PAGE) and subsequently transferred onto polyvinylidene fluoride (PVDF) membranes. After blocking with 5% non-fat milk in Tris-buffered saline containing 0.1% Tween 20 (TBST) for 1 h at room temperature, the membranes were incubated overnight at 4 °C with the following primary antibodies: anti-phospho-p38 (1:1000, Cat# AF4001, Affinity, Cincinnati, OH, USA), anti-p38 (1:5000, Cat# BF8015, Affinity, Cincinnati, OH, USA), and anti-GAPDH (1:1000, Cat# P30008S, Abmart, Shanghai, China). After being washed with TBST three times, the membranes were incubated for 1 h at room temperature with the corresponding horseradish peroxidase (HRP)-conjugated secondary antibodies, including anti-rabbit IgG (1:5000, Cat# M21002S, Abmart, Shanghai, China) and anti-mouse IgG (1:5000, Cat# M21001S, Abmart, Shanghai, China). The protein bands were visualized using an enhanced chemiluminescence (ECL) detection kit, and the relative protein expression levels were quantified via densitometric analysis using ImageJ software, with GAPDH serving as the internal control. For quantitative densitometry analysis, the band intensities of phosphorylated p38 (p-p38) MAPK and total p38 MAPK were first normalized to the internal loading control GAPDH. The activation status of the p38 MAPK pathway was subsequently determined by calculating the ratio of normalized p-p38 MAPK to normalized total p38 MAPK.

#### 4.2.8. Statistical Analysis

All studies were executed utilizing randomized and blinded protocols. Each in vitro assay was carried out with a minimum of three independent repetitions, and in vivo models contained at least three mice per assigned group (*n* ≥ 3). All quantitative data were expressed as the mean ± standard deviation (SD). Statistical analyses and data visualization were performed using GraphPad Prism software (version 9.5.0; GraphPad Software, San Diego, CA, USA). Differences among multiple groups were analyzed using a one-way analysis of variance (ANOVA), followed by Dunnett’s post hoc test. A *p*-value < 0.05 was considered statistically significant.

## 5. Conclusions

In conclusion, this study reveals the toxicological mechanism by which the environmental plasticizer DEHP exacerbates psoriasis injury through triggering local inflammatory responses, and demonstrates the targeted therapeutic potential of SH-NPs. The prepared SH-NPs effectively mitigate the DEHP-aggravated inflammatory microenvironment by targeted suppression of the p38 MAPK signaling pathway, inhibit keratinocyte hyperproliferation and reverse pathological tissue remodeling. Crucially, while exhibiting potent local anti-inflammatory efficacy comparable to that of traditional glucocorticoids, SH-NPs appear to avoid the severe macroscopic splenic atrophy typically associated with systemic immunosuppression. By elucidating this “toxicant–disease–drug” regulatory chain, our research highlights SH-NPs as a safe and highly efficacious targeted nanotherapy, providing robust experimental evidence and novel insights for managing complex inflammatory skin diseases exacerbated by environmental pollutants.

## Figures and Tables

**Table 1:** Primer sequences.

Gene Name	Forward Primer	Reverse Primer
TNF-α	TAGCCCACGTCGTAGCAAAC	TGTCTTTGAGATCCATGCCGT
IL-1β	TGCCACCTTTTGACAGTGATG	TGATGTGCTGCTGCGAGATT
β-actin	CGTTGACATCCGTAAAGACCTC	TAGGAGCCAGGGCAGTAATCT

## Data Availability

The datasets used or analyzed during the current study are available from the corresponding author upon reasonable request.

## Supplementary Materials

The following supporting information can be downloaded at: https://www.mdpi.com/article/10.3390/ijms27167109/s1.
