# Supplementary material for: Shikonin-Loaded Nanoparticles Ameliorate DEHP-Exacerbated Psoriasis Skin Injury via Targeted Inhibition of the p38 MAPK Signaling Pathway
Source: Int J Mol Sci. 2026 Aug 8;27(16):7109. doi: 10.3390/ijms27167109 (PMC13513280; doi:10.3390/ijms27167109)
Supplement: Supplementary file 1 [file ijms-27-07109-s001.zip › Supplementary Figure S1.pdf]

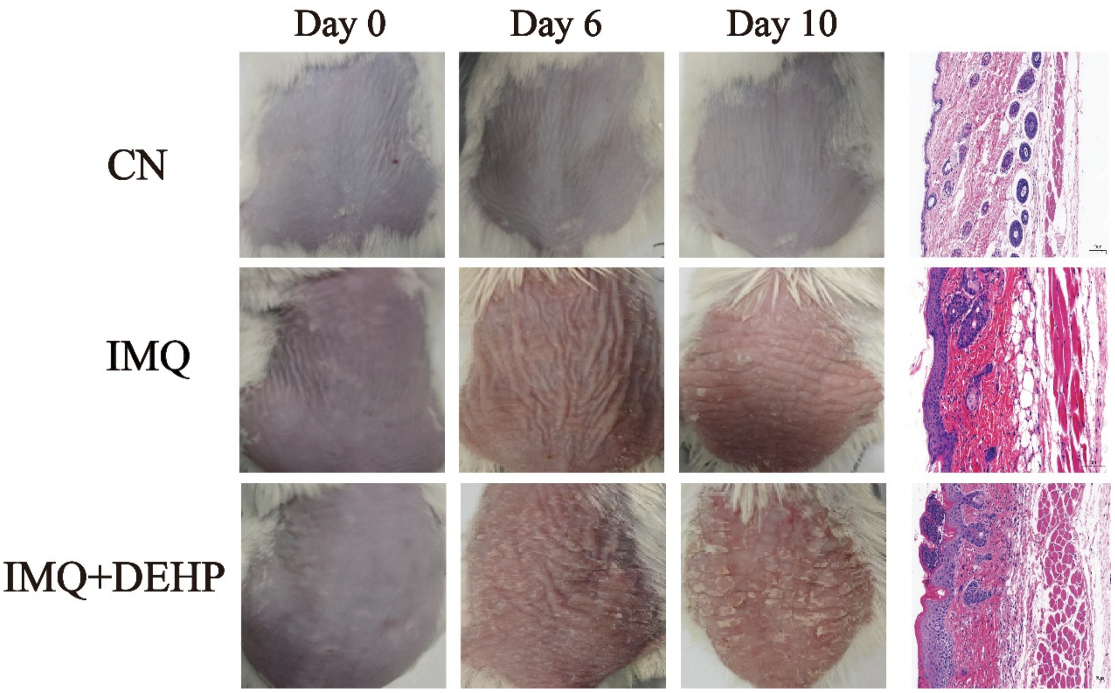

Supplementary Figure S1. DEHP exacerbates imiquimod (IMQ)-induced psoriasis-like skin lesions in mice. Representative macroscopic photographs and histological sections of the dorsal skin from the Control (CN), IMQ, and IMQ + DEHP groups. The left panels show the progression of gross morphological changes on Day 0, Day 6, and Day 10. While topical IMQ application induced visible erythema, scaling, and skin thickening compared to the CN group, co-exposure to DEHP (IMQ + DEHP) markedly aggravated these psoriasis-like symptoms. The rightmost panels display representative hematoxylin and eosin (H&E) staining of the dorsal skin tissues. Histological analysis further confirmed the exacerbating effect of DEHP, characterized by more severe epidermal hyperplasia (acanthosis), pronounced hyperkeratosis, and increased inflammatory cell infiltration in the dermis compared to the IMQ-only group.
